# Nonlinear Mechanical Property of 3D Braided Composites with Multi-Types Micro-Distortion: A Quantitative Evaluation

**DOI:** 10.3390/polym15061428

**Published:** 2023-03-14

**Authors:** Junjun Zhai, Xiangxia Kong, Luchen Wang, Shi Yan, Lili Jiang, Zhiwei Cai

**Affiliations:** 1 College of Aeronautics and Astronautics, North China Institute of Aerospace Engineering, Langfang 065000, China; 2 College of Civil Engineering and Architecture, Harbin University of Science and Technology, Harbin 150080, China; 3 Hebei Key Laboratory of Trans-Media Aerial Underwater Vehicle, North China Institute of Aerospace Engineering, Langfang 065000, China; 4 College of Material Engineering, North China Institute of Aerospace Engineering, Langfang 065000, China; 5 College of Civil Engineering and Architecture, Xiamen City University, Xiamen 361008, China

**Keywords:** carbon/resin composites, micro-distortion, mechanical properties, finite element analysis, 3D braiding

## Abstract

A new alternative calculation procedure is developed to quantify the effect of yarn distortion characteristics on the mechanical properties of three-dimensional (3D) braided carbon/resin composites. Firstly, the multi-type yarn distortion characteristics factors including path, cross-section shape and cross-section torsion effects are described based on the stochastic theory. Then, the multiphase finite element method is employed to overcome the complex discretization in traditional numerical analysis, and the parametric studies including multi-type yarn distortion and different braided geometrical parameters on the resulting mechanical properties are performed. It is shown that the proposed procedure can simultaneously capture the yarn path and cross-section distortion characteristics caused by the mutual squeeze of component materials, which is difficult to characterize by experimental methods. In addition, it is found that even small distortions of yarn may significantly affect the mechanical properties for 3D braided composites, and the 3D braided composites with different braiding geometric parameters will show different sensitivity to the distortion characteristics factors of yarn. The procedure, which could be implemented into commercial finite element codes, is an efficient tool for the design and structural optimization analysis of a heterogeneous material with anisotropic properties or complex geometries.

## 1. Introduction

Three-dimensional (3D) braided composites have been widely applied in aerospace and military fields due to their excellent mechanical performance. Since the 1980s, the research on 3D braided composites has received considerable attention by most developed countries, and a large amount of research on experimental, theoretical and numerical calculation approaches has been proposed [1,2,3,4,5]. Due to the complexity and periodic microstructure characteristics of 3D braided composites, most of the approaches are carried out based on the representative unit cell (RUC). Frank et al. [6] first proposed the term fiber structure, and a meter-shaped RUC model that can reflect the forming process for braided composites was presented. Wang et al. [7] determined an internal fiber topology model of the 3D braided preform by tracking the movement of the yarn carrier in the braided preform process. Wu et al. [8] proposed a three cell model composed of panel elements on repeatable boundaries, column elements on corners and internal primitives based on four-step forming, which was adopted by the majority of research scholars. Then, Chen et al. [9], Zheng et al. [10] and Li et al. [11] proposed four different RUC models by modifying the mathematical relationship between the internal structural of the three cell model for 3D braided composites. Zeng et al. [12] reported a helix geometry RUC model by considering the regular torsion and squeeze of the braided yarn. Li et al. [13] and Zhang et al. [14] simplified the section shape into an ellipse, established the new RUC models and described the micro-structure more accurately. In addition, some researchers [15,16,17] have also considered the cross-section squeezing deformation of braided yarn and simplified the distorted cross-section as a polygon. Zhu et al. [18] and Wang et al. [19] considered the squeeze deformation of braided yarn caused by a tightening process, and established an improved RUC model in which the cross-section shape changes continuously along the axial direction of the yarn. With the continuous improvement of modeling approaches, more and more available models [20,21] close to the real micro-structure for 3D braided composites have been developed.

In practice, the distortion of yarn morphology caused by yarn squeeze is a real physical phenomenon and exhibits some certain random characteristics. Up to now, the influence of random yarn characteristics on the mechanical properties of fiber-reinforced composites has been a concern of some scholars [22,23,24,25,26,27,28]. Yushanov and Bogdanovich [22,23] developed an analytical method and analyzed the effects of random waviness of reinforcements on the elastic constants of unidirectional, biaxial and orthogonally woven composites. Amato et al. [24] studied the nonlinear mechanical behavior of fabric composites caused by the change of yarn variation by a finite element method. Kang et al. [25] investigated the effects of yarn torsion on the mechanical properties of 3D woven composites and pointed out that irregular yarn had a significant effect on the elastic properties and axial tensile strength of the yarn. Adumitroaie et al. [26] found that the elastic constants of 2D orthogonal woven composites are related to yarn spacing and fluctuation and that the change of elastic constants will affect the failure load and initial failure position. Wang et al. [27] proposed a variable theory of elastic constants of textile composites with random yarn geometry, in which the influence of yarn cross-section fluctuation on the elastic constants of textile composites was studied. Fang et al. [28] analyzed the influence of irregular yarns on elastic properties of 3D braided carbon/resin composites based on the stochastic theory, in which the cross-section of each braid yarn is set as an octagon and divided into seven regions in the RUC. These great results are of great significance to improve the prediction accuracy of mechanical responses for fiber-reinforced composites.

To sum up, the view that yarn morphology will affect the mechanical properties of fiber-reinforced composites has been pointed out by some scholars, but yarn distortion is a manufacturing defect for 3D braided composites and cannot be avoided. The change of mechanical properties of 3D braided composites caused by yarn distortion is likely to make it difficult for researchers to determine the main factors that lead to the failure load and initial failure position for the overall structure. However, the research about the effect of random distortion morphology of reinforcing yarn on the mechanical properties of 3D braided composites is relatively small at present; the relationship between mechanical properties and yarn distortion characteristic parameters is still not clear and is difficult to control. In view of the current situation, where the structural design of 3D braided composites tends to be optimized and the use of large/complex special-shaped components is gradually increasing, it is necessary to develop a new calculation method to quantify the variation law of mechanical properties with yarn distortion for 3D braided composites. This is of great significance to the assessment of the real strength margin of large 3D braided composite structures in the future.

In this work, the fluctuation characteristics of mechanical properties for 3D braided carbon/resin composites caused by yarn distortion are mainly studied, and the analyses are performed based on the microscopic RUC scale. The distortion morphology of reinforced yarn is mainly expressed by three kinds of parameters: yarn path, yarn cross-section size and angular torsion; the randomness of each parameter is characterized in 3D space by its mean and covariance matrix. Due to the complex geometry of 3D braided carbon/resin composites, the resin matrix is assumed to be isotropic and the yarn is regarded as a unidirectional composite which has transversely isotropic elastic properties; the multiphase finite element method is employed to overcome the difficulty of discretization for the yarn and matrix in traditional numerical analysis. To demonstrate the efficiency of the proposed scheme, the influence of yarn distortion parameters on its mechanical properties is presented first. It is shown that the improved finite element calculation procedure can simultaneously capture the yarn path and cross-section distortion characteristics caused by the mutual squeeze of component materials, which is difficult to characterize by experimental methods. Based upon the proposed calculation scheme, the parametric studies including the distortion characteristics of yarns and braided geometrical parameters of 3D braided carbon/resin composites are performed to obtain the effect law on the resulting mechanical properties, and some valuable conclusions are obtained. The advantage of this procedure is that an exact description of the distortion characteristics for yarns and complex discretization for RUC are not required for predicting mechanical properties of braided carbon/resin composites with stochastic geometries or anisotropic properties. The procedure, which could be implemented into commercial finite element codes, is an efficient tool for the design and structural optimization analysis of a heterogeneous material with anisotropic properties or complex geometries.

## 2. Characteristic Parameters and Properties of Distortion Yarn

### 2.1. Introduction of Characteristic Parameters

**Figure 1:**
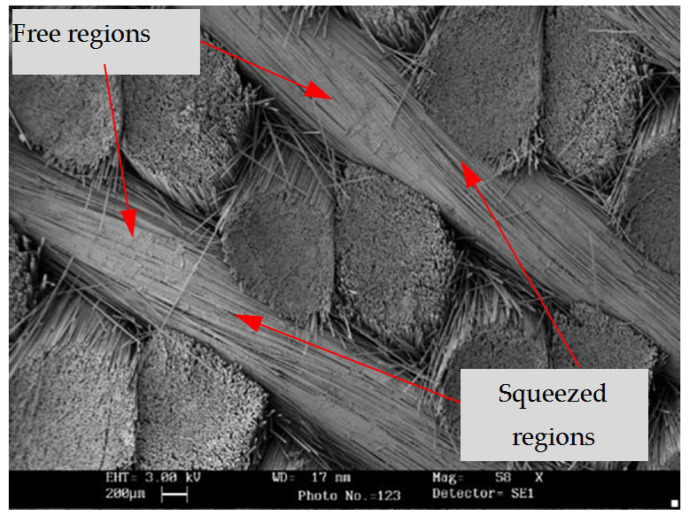
The SEM micrographs of 3D four-directional braided composites [9].

As shown in Figure 1 [9], yarn distortion is a manufacturing defect for 3D braided composites and cannot be avoided; the braided yarn becomes more irregular and shows random characteristics due to the mutual squeeze, although the shape of the braided yarn is generally considered to be regular before the yarn is braided into a preform. The irregularity of yarn includes the random fluctuation of the yarn path, the random scaling of the yarn cross-section shape and the random cross-section torsion. In order to simplify the problem, it is assumed that three types of characteristic parameters considering yarn distortion are independent of each other, the fiber volume fraction on each yarn section is constant and that all yarns in a RUC have the same statistical properties.

**Figure 2:**
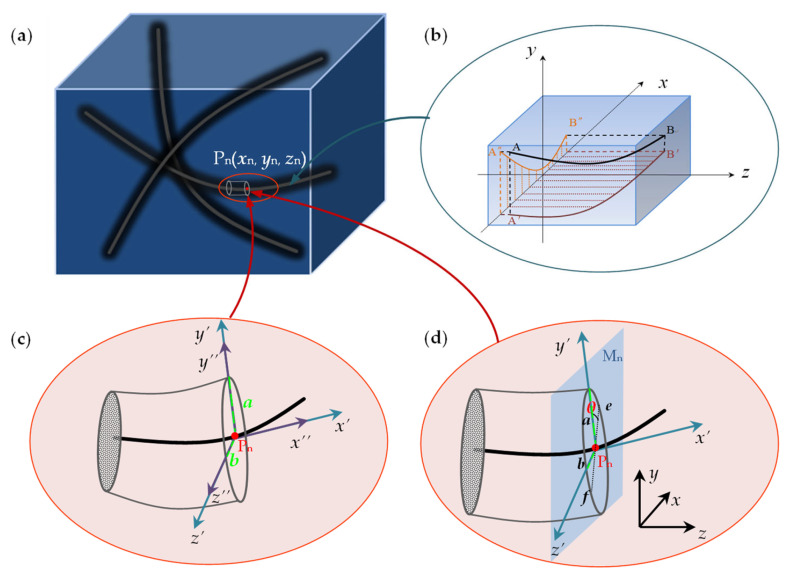
Characteristic parameters and coordinate system of distortion yarn: (**a**) multidirectional yarn reinforcement structure frame, (**b**) reinforcement path, (**c**) cross-section scaling, (**d**) cross-section torsion.

In order to describe the random parameters above, consider the solid structure with multidirectional yarn reinforcement shown in Figure 2a. Each yarn occupies some volume in the 3D space without penetrating into other yarns. Thus, each yarn can be treated separately. An individual yarn shown in Figure 2b is characterized by the centerline AB. In order to describe the random parameters of the yarn above, a set of orthogonal coordinate axes composed of *x*–*y*–*z* are defined as the reinforcement coordinate system for reinforcement yarn, where the *x*-axis is longitudinal along the yarn. Then, the yarn path vector r in the reinforcement coordinate system can be described by three arbitrary position parameters (*x*_n_, *y*_n_, *z*_n_) on the center path of the yarn, as shown in Figure 2a. In addition, the distortion characteristics of the yarn path can be decomposed into the random fluctuations of the projection of the path vector in the *x*–*y* and *x*–*z* planes, as shown in Figure 2b.

In order to describe the distortion characteristics of the cross-sectional shape of the yarn, the cross-section shape is assumed to be elliptical, and the cross-section is perpendicular to the tangent direction *x*’ of the yarn path at the point, as shown in Figure 2c. The parameters *a* and *b* represent the semi-major axes of the elliptical cross-section, and the local coordinate systems *y*’ and *z*’ are along the two semi-major axes of the cross-section, respectively; then, the random scaling of the shape parameters (*a*, *b*) can be used to describe the random distortion characteristics of the cross-section shape for the braided yarn.

The irregular torsional characteristics of the elliptical cross-section of the yarn are also considered. Suppose that there is a plane $M_{n}$ parallel to the *x*–*y* plane of the reinforcement coordinate system and passing through any point $P_{n}$ on the path of the yarn, and the intersection line of the plane and the cross-section corresponding to the yarn at point $P_{n}$ is recorded as ef. As shown in Figure 2d, the angle θ between the yarn’s semi-major axis and the intersection line ef is defined as the cross-sectional torsional angle, and the random torsional characteristics of the cross-section for the yarn can be characterized by describing the random change of the torsional angle θ.

### 2.2. Expansion of Characteristic Parameters

In order to describe the randomness of the distortion to the yarn path and cross-section caused by yarn squeeze uncertainty, according to the representation method of canonical expansion of any random function [29], the projection of random path vector in *x*–*y* and *x*–*z* planes, cross-section size (*a*, *b*) or cross-section torsion angle θ can be expressed in the form of a pure deterministic component and a pure random component.

(1) $${ℜ}_{i}\left(\xi \right)=\Phi_{0}^{i}\left(\xi \right)+\sum_{k=1}^{\infty }V_{k}^{i}\varphi_{k}^{i}\left(\xi \right),i=L,F,N$$

where L represents the projection of the path vector in the *x*–*y* and *x*–*z* planes, F represents the cross-section size, N is the cross-section torsion angle, $\Phi_{0}^{i}\left(\xi \right)$ is the specified deterministic function, $\left\{\varphi_{k}^{i}\left(\xi \right)\right\}$ is the deterministic coordinate basis function, $\left\{V_{k}^{i}\right\}$ is the set of orthogonal zero-mean random values and ξ is the running parameter.

Therefore, the randomness of ${ℜ}_{i}\left(\xi \right)$ is completely determined by the distribution density of the coefficient $V_{k}^{i}$. In practical application, it is more convenient to adopt the numerical characteristic of $V_{k}^{i}$ than the distribution density. The basic properties of common random functions can be characterized by its first-order moment and second-order moment. The definition of moment is as follows:

(2) $$\langle {ℜ}_{i}\left(\xi \right)\rangle =\Phi_{0}^{i}\left(\xi \right)$$

(3) $${\hat{K}}_{{ℜ}_{i}{ℜ}_{i}}\left(\xi ,\zeta \right)=\langle \overset{\circ }{{ℜ}_{i}}\left(\xi \right)⊗\overset{\circ }{{ℜ}_{i}}\left(\xi +\zeta \right)\rangle =\sum_{k=1}^{\infty }\langle V_{k}^{i}⊗V_{k}^{i}\rangle \varphi_{k}^{i}\left(\xi \right)\varphi_{k}^{i}\left(\xi +\zeta \right)$$

where ${\overset{\circ }{ℜ}}_{i}\left(\xi \right)={ℜ}_{i}\left(\xi \right)-\langle {ℜ}_{i}\left(\xi \right)\rangle$ represents a centered random function, $\langle {ℜ}_{i}\left(\xi \right)\rangle$ is called the mean of ${ℜ}_{i}\left(\xi \right)$ and the second central moment ${\hat{K}}_{{ℜ}_{i}{ℜ}_{i}}\left(\xi ,\zeta \right)$ is the covariance function.

### 2.3. Theoretical Derivation

The randomness of the path, cross-section size and torsion angle caused by a squeeze of the yarn is described by the randomness of local basis vector corresponding to each point on the path. The unit vectors in each direction of the reinforcement coordinate system *x*–*y*–*z* are written as $\varepsilon_{1}$, $\varepsilon_{2}$, $\varepsilon_{3}$; according to the definition of the local coordinate system in Figure 2c, the unit vector $\varepsilon_{1}^{\prime }$ of the local coordinate system corresponding to any point of the path along the *x*’ direction can be defined as

(4) $$\varepsilon_{1}^{\prime }\;=\;\;\dot{r}\left(\xi \right)/\left|\dot{r}\left(\xi \right)\right|$$

According to the geometric relationship and vector calculation method of yarn cross-section torsion angle, reinforcement coordinate system and local coordinate system defined in Figure 2, the unit vector $\varepsilon_{2}^{\prime }$ along the semi-major axis *y*’ of the elliptical cross-section can be obtained through Rodrigues’ rotation formula as follows:

(5) $$\varepsilon_{2}^{\prime }=\left\{\left(\varepsilon_{3}\times \varepsilon_{1}^{\prime }\right)\cos\theta +\left[\varepsilon_{1}^{\prime }\times \left(\varepsilon_{3}\times \varepsilon_{1}^{\prime }\right)\right]\sin\theta \right\}/\left|\varepsilon_{3}\times \varepsilon_{1}^{\prime }\right|$$

Therefore, along the semi-minor axis *z*’ direction of the elliptical cross-section, the unit vector $\varepsilon_{3}^{\prime }$ can be obtained by the following vector calculation.

(6) $$\varepsilon_{3}^{\prime }=\varepsilon_{1}^{\prime }\times \varepsilon_{2}^{\prime }=\left\{\left[\varepsilon_{1}^{\prime }\times \left(\varepsilon_{3}\times \varepsilon_{1}^{\prime }\right)\right]\cos\theta -\left(\varepsilon_{3}\times \varepsilon_{1}^{\prime }\right)\sin\theta \right\}/\left|\varepsilon_{3}\times \varepsilon_{1}^{\prime }\right|$$

Although the local unit vector $\varepsilon_{1}^{\prime }$, $\varepsilon_{2}^{\prime }$, $\varepsilon_{3}^{\prime }$ at any point of the path can be used to represent the irregularity of the yarn path and cross-sectional torsion angle as described in Section 2.2, the relevant information of yarn cross-sectional size distortion cannot be expressed well. Therefore, an additional set of coordinate system *x*’’–*y*’’–*z*’’ (as shown in Figure 2c) for local cross-sectional size scaling, which completely coincides with the local coordinate system *x*’–*y*’–*z*’, is established. However, the scale of the base vector between the local coordinate system and the scaling coordinate system is different. In order to establish the transformation relationship between the base vectors $\varepsilon_{1}^{\prime \prime }$, $\varepsilon_{2}^{\prime \prime }$, $\varepsilon_{3}^{\prime \prime }$ of the random scaling coordinate system and the base vectors $\varepsilon_{1}^{\prime }$, $\varepsilon_{2}^{\prime }$, $\varepsilon_{3}^{\prime }$ of the local coordinate system, a set of scaling matrices $\hat{\vartheta }{}^{\prime \prime }\left(\xi \right)$ are introduced.

(7) $$\hat{\vartheta }{}^{\prime \prime }\left(\xi \right)=diag\left\{\vartheta_{11}{}^{\prime \prime }\left(\xi \right),\vartheta_{22}{}^{\prime \prime }\left(\xi \right),\vartheta_{33}{}^{\prime \prime }\left(\xi \right)\right\}=diag\left\{1,\frac{a\left(\xi \right)}{\tilde{a}},\frac{b\left(\xi \right)}{\tilde{b}}\right\}$$

(8) $$\varepsilon_{1}^{\prime \prime }=\hat{\vartheta }{}^{\prime \prime }\cdot \varepsilon_{1}^{\prime },\;\varepsilon_{2}^{\prime \prime }=\hat{\vartheta }{}^{\prime \prime }\cdot \varepsilon_{2}^{\prime },\;\varepsilon_{3}^{\prime \prime }=\hat{\vartheta }{}^{\prime \prime }\cdot \varepsilon_{3}^{\prime }$$

where the elements in the scaling matrices $\hat{\vartheta }{}^{\prime \prime }\left(\xi \right)$ represent the scaling factors along each coordinate axis and $\tilde{a}=\langle a\left(\xi \right)\rangle ,\;\tilde{b}=\langle b\left(\xi \right)\rangle$.

Obviously, the local random scaling basis vectors $\varepsilon_{1}^{\prime \prime }$, $\varepsilon_{2}^{\prime \prime }$, $\varepsilon_{3}^{\prime \prime }$ are the first derivative of the path vector and a function of the scaling matrix and the torsion angle. In order to obtain the mean and covariance of local basis vectors related to the random reinforcement path, cross-section torsion and cross-section scaling, the Taylor expansion of the basis vectors $\varepsilon_{1}^{\prime \prime }$, $\varepsilon_{2}^{\prime \prime }$, $\varepsilon_{3}^{\prime \prime }$ is employed along the mean parameters $\dot{r}=\langle \dot{r}\rangle$,

(9) $$\begin{array}{l} \hat{\vartheta }{}^{\prime \prime }=\langle \hat{\vartheta }{}^{\prime \prime }\rangle ,\;\theta =\langle \theta \rangle .\\ \;\;\;\varepsilon_{i}^{\prime \prime }={\left.\varepsilon_{i}^{\prime \prime }\right|}_{{}_{\begin{array}{l} \theta =\langle \theta \rangle \\ \dot{r}=\langle \dot{r}\rangle \\ \hat{\vartheta }{}^{\prime \prime }=\langle \hat{\vartheta }{}^{\prime \prime }\rangle \end{array}}}+{\left.\frac{\partial \varepsilon_{i}^{\prime \prime }}{\partial \dot{r}}\right|}_{\begin{array}{l} \theta =\langle \theta \rangle \\ \dot{r}=\langle \dot{r}\rangle \\ \hat{\vartheta }{}^{\prime \prime }=\langle \hat{\vartheta }{}^{\prime \prime }\rangle \end{array}}\cdot \overset{\circ }{\dot{r}}+{\left.\frac{\partial \varepsilon_{i}^{\prime \prime }}{\partial \theta }\right|}_{\begin{array}{l} \theta =\langle \theta \rangle \\ \dot{r}=\langle \dot{r}\rangle \\ \hat{\vartheta }{}^{\prime \prime }=\langle \hat{\vartheta }{}^{\prime \prime }\rangle \end{array}}\overset{\circ }{\theta }+\\ \;\;\;\;\;\;\;\;\;{\left.\frac{\partial \varepsilon_{i}^{\prime \prime }}{\partial \hat{\vartheta }{}^{\prime \prime }}\right|}_{\begin{array}{l} \theta =\langle \theta \rangle \\ \dot{r}=\langle \dot{r}\rangle \\ \hat{\vartheta }{}^{\prime \prime }=\langle \hat{\vartheta }{}^{\prime \prime }\rangle \end{array}}:\overset{\circ }{\hat{\vartheta }{}^{\prime \prime }}+\frac{1}{2}{\left.\frac{\partial^{2}\varepsilon_{i}^{\prime \prime }}{\partial \dot{r}\partial \dot{r}}\right|}_{\begin{array}{l} \theta =\langle \theta \rangle \\ \dot{r}=\langle \dot{r}\rangle \\ \hat{\vartheta }{}^{\prime \prime }=\langle \hat{\vartheta }{}^{\prime \prime }\rangle \end{array}}:\overset{\circ }{\dot{r}}⊗\overset{\circ }{\dot{r}}+\frac{1}{2}{\left.\frac{\partial^{2}{\varepsilon^{\prime \prime }}_{i}}{\partial \theta \partial \theta }\right|}_{\begin{array}{l} \theta =\langle \theta \rangle \\ \dot{r}=\langle \dot{r}\rangle \\ \hat{\vartheta }{}^{\prime \prime }=\langle \hat{\vartheta }{}^{\prime \prime }\rangle \end{array}}\cdot \left(\overset{\circ }{\theta }\cdot \overset{\circ }{\theta }\right)+\ldots \ldots \end{array}$$

where the second and fifth items reflect the random fluctuation of the path, the third and sixth items define the random cross-section torsion of the yarn and the fourth item reflects the random scaling of the yarn cross-section.

Therefore, the second-order approximation of the mean of locally randomly scaled basis vectors can be expressed as

(10) $$\langle \varepsilon_{i}^{\prime \prime }\rangle ={\left.\varepsilon_{i}^{\prime \prime }\right|}_{{}_{\begin{array}{l} \theta =\langle \theta \rangle \\ \dot{r}=\langle \dot{r}\rangle \\ \hat{\vartheta }{}^{\prime \prime }=\langle \hat{\vartheta }{}^{\prime \prime }\rangle \end{array}}}+\frac{1}{2}{\left.\frac{\partial^{2}\varepsilon_{i}^{\prime \prime }}{\partial \dot{r}\partial \dot{r}}\right|}_{\begin{array}{l} \theta =\langle \theta \rangle \\ \dot{r}=\langle \dot{r}\rangle \\ \hat{\vartheta }{}^{\prime \prime }=\langle \hat{\vartheta }{}^{\prime \prime }\rangle \end{array}}:{\hat{K}}_{\dot{r}\dot{r}}+\frac{1}{2}{\left.\frac{\partial^{2}\varepsilon_{i}^{\prime \prime }}{\partial \theta \partial \theta }\right|}_{\begin{array}{l} \theta =\langle \theta \rangle \\ \dot{r}=\langle \dot{r}\rangle \\ \hat{\vartheta }{}^{\prime \prime }=\langle \hat{\vartheta }{}^{\prime \prime }\rangle \end{array}}\cdot K_{\theta \theta }$$

where ${\hat{K}}_{\dot{r}\dot{r}}=diag\left\{0,K_{\dot{y}\dot{y}},K_{\dot{z}\dot{z}}\right\}=K_{\dot{h}\dot{h}}\varepsilon ⊗\varepsilon$, $K_{\dot{h}\dot{h}}=\langle \overset{\circ }{\dot{h}}\overset{\circ }{\dot{h}}\rangle \approx \sigma_{\alpha_{i}}^{2}$,$\sigma_{\alpha_{i}}^{2}$ is the standard deviation of the deflection angle of the yarn path in plane *x*–*y* and plane *x*–*z*, which is defined as the angle between the ideal yarn path direction and the local tangent vector of the random yarn path. In addition, $K_{\theta \theta }=\langle \overset{\circ }{\theta }\overset{\circ }{\theta }\rangle =\sigma_{\theta }^{2}$, $\sigma_{\theta }$ is the standard deviation of the cross-section torsion angle θ.

The centralized local random scaling basis vector $\overset{\circ }{\varepsilon_{i}^{\prime \prime }}$ and the first-order approximation of the covariance can be expressed as Equations (11) and (12), respectively.

(11) $$\overset{\circ }{\varepsilon_{i}^{\prime \prime }}={\left.\frac{\partial \varepsilon_{i}^{\prime \prime }}{\partial \dot{r}}\right|}_{\begin{array}{l} \theta =\langle \theta \rangle \\ \dot{r}=\langle \dot{r}\rangle \\ \hat{\vartheta }{}^{\prime \prime }=\langle \hat{\vartheta }{}^{\prime \prime }\rangle \end{array}}\cdot \overset{\circ }{\dot{r}}+{\left.\frac{\partial \varepsilon_{i}^{\prime \prime }}{\partial \hat{\vartheta }{}^{\prime \prime }}\right|}_{\begin{array}{l} \theta =\langle \theta \rangle \\ \dot{r}=\langle \dot{r}\rangle \\ \hat{\vartheta }{}^{\prime \prime }=\langle \hat{\vartheta }{}^{\prime \prime }\rangle \end{array}}:\overset{\circ }{\hat{\vartheta }{}^{\prime \prime }}+{\left.\frac{\partial \varepsilon_{i}^{\prime \prime }}{\partial \theta }\right|}_{\begin{array}{l} \theta =\langle \theta \rangle \\ \dot{r}=\langle \dot{r}\rangle \\ \hat{\vartheta }{}^{\prime \prime }=\langle \hat{\vartheta }{}^{\prime \prime }\rangle \end{array}}\overset{\circ }{\theta }$$

(12) $$\begin{array}{l} \langle \overset{\circ }{\varepsilon_{i}^{\prime \prime }}⊗\overset{\circ }{\varepsilon_{j}^{\prime \prime }}\rangle ={\left.\frac{\partial \varepsilon_{i}^{\prime \prime }}{\partial \dot{r}}\right|}_{\begin{array}{l} \theta =\langle \theta \rangle \\ \dot{r}=\langle \dot{r}\rangle \\ \hat{\vartheta }{}^{\prime \prime }=\langle \hat{\vartheta }{}^{\prime \prime }\rangle \end{array}}\cdot {\hat{K}}_{\dot{r}\dot{r}}\cdot {\left({\left.\frac{\partial \varepsilon_{j}^{\prime \prime }}{\partial \dot{r}}\right|}_{\begin{array}{l} \theta =\langle \theta \rangle \\ \dot{r}=\langle \dot{r}\rangle \\ \hat{\vartheta }{}^{\prime \prime }=\langle \hat{\vartheta }{}^{\prime \prime }\rangle \end{array}}\right)}^{T}+\\ \;\;\;\;\;\;\;\;\;\;\;\;\;\;\;\;\;\;\;{\left.\frac{\partial \varepsilon_{i}^{\prime \prime }}{\partial \hat{\vartheta }{}^{\prime \prime }}\right|}_{\begin{array}{l} \theta =\langle \theta \rangle \\ \dot{r}=\langle \dot{r}\rangle \\ \hat{\vartheta }{}^{\prime \prime }=\langle \hat{\vartheta }{}^{\prime \prime }\rangle \end{array}}:K_{\hat{\vartheta }{}^{\prime \prime }\hat{\vartheta }{}^{\prime \prime }}:{\left({\left.\frac{\partial \varepsilon_{j}^{\prime \prime }}{\partial \hat{\vartheta }{}^{\prime \prime }}\right|}_{\begin{array}{l} \theta =\langle \theta \rangle \\ \dot{r}=\langle \dot{r}\rangle \\ \hat{\vartheta }{}^{\prime \prime }=\langle \hat{\vartheta }{}^{\prime \prime }\rangle \end{array}}\right)}^{T}+K_{\theta \theta }{\left.\frac{\partial \varepsilon_{i}^{\prime \prime }}{\partial \theta }\right|}_{\begin{array}{l} \theta =\langle \theta \rangle \\ \dot{r}=\langle \dot{r}\rangle \\ \hat{\vartheta }{}^{\prime \prime }=\langle \hat{\vartheta }{}^{\prime \prime }\rangle \end{array}}⊗{\left.\frac{\partial \varepsilon_{j}^{\prime \prime }}{\partial \theta }\right|}_{\begin{array}{l} \theta =\langle \theta \rangle \\ \dot{r}=\langle \dot{r}\rangle \\ \hat{\vartheta }{}^{\prime \prime }=\langle \hat{\vartheta }{}^{\prime \prime }\rangle \end{array}} \end{array}$$

For a scaling matrix $\hat{\vartheta }{}^{\prime \prime }$, its covariance function $K_{\hat{\vartheta }{}^{\prime \prime }\hat{\vartheta }{}^{\prime \prime }}$ in Equation (12) is defined as follows, and the random fluctuations of scaling factors along the orthogonal axis *y*’’–*z*’’ have corresponding covariance functions.

(13) $$K_{\hat{\vartheta }{}^{\prime \prime }\hat{\vartheta }{}^{\prime \prime }}=\langle \overset{\circ }{\hat{\vartheta }{}^{\prime \prime }}⊗\overset{\circ }{\hat{\vartheta }{}^{\prime \prime }}\rangle =K_{\vartheta_{jk}{}^{\prime \prime }\vartheta_{lm}{}^{\prime \prime }}\varepsilon_{j}⊗\varepsilon_{k}⊗\varepsilon_{l}⊗\varepsilon_{m}$$

(14) $$\begin{array}{l} K_{\vartheta_{22}{}^{\prime \prime }\vartheta_{22}{}^{\prime \prime }}=\langle \overset{\circ }{\vartheta_{22}{}^{\prime \prime }}\overset{\circ }{\vartheta_{22}{}^{\prime \prime }}\rangle =\langle \overset{\circ }{a}\overset{\circ }{a}\rangle /{\tilde{a}}^{2}={\left(\sigma_{a}/\tilde{a}\right)}^{2}=c_{a}^{2}\\ K_{\vartheta_{33}{}^{\prime \prime }\vartheta_{33}{}^{\prime \prime }}=\langle \overset{\circ }{\vartheta_{33}{}^{\prime \prime }}\overset{\circ }{\vartheta_{33}{}^{\prime \prime }}\rangle =\langle \overset{\circ }{b}\overset{\circ }{b}\rangle /{\tilde{b}}^{2}={\left(\sigma_{b}/\tilde{b}\right)}^{2}=c_{b}^{2} \end{array}$$

where $c_{a}$ and $c_{b}$ represent the coefficient of variation along the *y*’ and *z*’ directions, respectively.

Based on the above description, the following relationship between the material property matrix tensor $\mathbb{N}{}^{\prime \prime }$ of yarn under the current random scaling coordinate system *x*’’–*y*’’–*z*’’ and the material property matrix tensor ℕ of yarn under the reinforcement coordinate system *x–y–z* can be established.

(15) $${\mathbb{N}}_{ijkl}={\mathbb{N}}_{mnop}^{"\prime \prime }\vartheta_{mr}^{\prime \prime }\varepsilon_{ri}^{\prime }\vartheta_{ns}^{\prime \prime }\varepsilon_{sj}^{\prime }\vartheta_{ot}^{\prime \prime }\varepsilon_{tk}^{\prime }\vartheta_{pu}^{\prime \prime }\varepsilon_{ul}^{\prime }$$

where $\varepsilon_{ji}^{\prime }=\varepsilon_{j}^{\prime }\cdot \varepsilon_{i}$ represents the directional cosine between the local unit vector and the unit vector of the reinforcement coordinate system.

Then, the variable scale cosine vector $\beta_{ij}^{\prime \prime }=\vartheta_{ir}^{\prime \prime }\varepsilon_{rj}^{\prime }$ is defined based on Equations (8) and (15), and Equation (15) can be written as

(16) $${\mathbb{N}}_{ijkl}={\mathbb{N}}_{mnop}^{\prime \prime }\beta_{mi}^{\prime \prime }\beta_{nj}^{\prime \prime }\beta_{ok}^{\prime \prime }\beta_{pl}^{\prime \prime }$$

It can be found that the material property matrix vector ℕ of yarn in the reinforcement coordinate system is a random function of the variable scale cosine vector β; the mean value material property matrix $\langle \mathbb{N}\rangle$ of yarn can be obtained by Taylor expansion of the random vector ℕ with respect to the variable scale cosine vector $\beta^{\prime \prime }=\langle \beta^{\prime \prime }\rangle$.

(17) $$\begin{array}{l} \mathbb{N}=\mathbb{N}\left|{}_{\beta^{\prime \prime }=\langle \beta^{\prime \prime }\rangle }\right.+\frac{\partial \mathbb{N}}{\partial \beta^{\prime \prime }}\left|{}_{\beta^{\prime \prime }=\langle \beta^{\prime \prime }\rangle }\right.\cdot \left(\beta^{\prime \prime }-\langle \beta^{\prime \prime }\rangle \right)+\\ \;\;\;\;\;\;\frac{1}{2}\frac{\partial^{2}\mathbb{N}}{\partial \beta^{\prime \prime }\partial \beta^{\prime \prime }}\left|{}_{\beta^{\prime \prime }=\langle \beta^{\prime \prime }\rangle }\right.:\left(\beta^{\prime \prime }-\langle \beta^{\prime \prime }\rangle \right)⊗\left(\beta^{\prime \prime }-\langle \beta^{\prime \prime }\rangle \right) \end{array}$$

(18) $$\langle \mathbb{N}\rangle ={\left.\mathbb{N}\right|}_{\beta^{\prime \prime }=\langle \beta^{\prime \prime }\rangle }+\frac{1}{2}\frac{\partial^{2}\mathbb{N}}{\partial \beta^{\prime \prime }\partial \beta^{\prime \prime }}\left|{}_{\beta^{\prime \prime }=\langle \beta^{\prime \prime }\rangle }\right.:{\hat{K}}_{\beta^{\prime \prime }\beta^{\prime \prime }}$$

Because the mechanical properties of yarn can be regarded as transversally isotropic, there are five independent elastic constants: $E_{11}^{\prime \prime }$, $E_{22}^{\prime \prime }$, $G_{12}^{\prime \prime }$, $v_{12}^{\prime \prime }$, $v_{23}^{\prime \prime }$, which can be obtained as follows:

(19) $$\begin{array}{l} E_{11}^{\prime \prime }=V_{f}E_{f11}+\left(1-V_{f}\right)E_{m}\\ E_{22}^{\prime \prime }=E_{33}^{\prime \prime }=E_{m}/\left(1-\sqrt{V_{f}}\left(1-E_{m}/E_{f22}\right)\right)\\ G_{12}^{\prime \prime }=G_{13}^{\prime \prime }=G_{m}/\left(1-\sqrt{V_{f}}\left(1-G_{m}/G_{f12}\right)\right)\\ G_{23}^{\prime \prime }=G_{m}/\left(1-\sqrt{V_{f}}\left(1-G_{m}/G_{f23}\right)\right)\\ v_{12}^{\prime \prime }=v_{13}^{\prime \prime }=V_{f}v_{f12}+\left(1-V_{f}\right)v_{m}\\ v_{23}^{\prime \prime }=E_{22}^{\prime \prime }/\left(2G_{23}^{\prime \prime }\right)-1 \end{array}$$

where the subscript *f* denotes fiber and *m* denotes matrix, while the parameters with superscript ″ denote the elastic constants of ideal yarn.

The relationship between elastic constants and compliance in the material property matrix can be represented as

(20) $$\begin{array}{l} S_{11}^{\prime \prime }=\frac{1}{E_{11}^{\prime \prime }},S_{12}^{\prime \prime }=-\frac{v_{12}^{\prime \prime }}{E_{22}^{\prime \prime }},S_{13}^{\prime \prime }=-\frac{v_{13}^{\prime \prime }}{E_{33}^{\prime \prime }}\\ S_{22}^{\prime \prime }=\frac{1}{E_{22}^{\prime \prime }},S_{23}^{\prime \prime }=-\frac{v_{23}^{\prime \prime }}{E_{33}^{\prime \prime }},S_{33}^{\prime \prime }=-\frac{1}{E_{33}^{\prime \prime }}\\ S_{44}^{\prime \prime }=\frac{1}{G_{23}^{\prime \prime }},S_{55}^{\prime \prime }=-\frac{1}{G_{13}^{\prime \prime }},S_{66}^{\prime \prime }=\frac{1}{G_{12}^{\prime \prime }} \end{array}$$

Finally, the relationship between the mean material property matrix $\langle \mathbb{N}\rangle$ of yarn and three kinds of random characteristic parameters $\sigma_{\alpha_{i}}\left(i=x-y,x-z\right)$, $c_{i}$$\left(i=a,b\right)$ and $\sigma_{\theta }$ in the reinforcement coordinate system is established, and the mechanical properties of distortion yarns can be effectively calculated.

**Figure 3:**
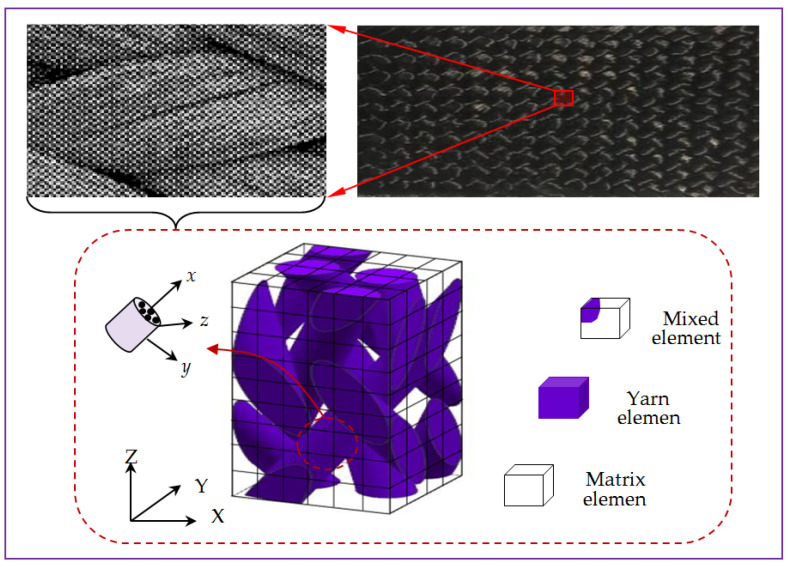
Microscopic representative unit cell model and three kinds of sub-cells.

According to the fiber braiding process, the yarns are straightened and deformed during the tightening process. Following microscopic observation, the following assumptions are proposed: (1) the yarns are straightened; (2) the interface between the yarn and matrix is in good contact, and the interface effect is not considered. Based on the topological relation and the shape of the cross section, the RUC model of 3D braided carbon/resin composites is established as shown in Figure 3 [11]. The symbols X–Y–Z in capital form represent the coordinate system under the overall structure of 3D braided composites. Symbol Z represents the longitudinal direction and symbols X–Y represent the transverse direction. In order to overcome the difficulty of discretization for the yarn and matrix of 3D braided carbon/resin composites in numerical analysis, the multiphase finite element method [12] is employed. The micro-scale RUC is divided into three kinds of 20-node rectangular isoparametric elements including the yarn element containing only the yarns, the matrix element containing only the resin and the mixed element containing both the yarn and the resin, and 27 Gauss integral points are selected in each kind of element to calculate the total compliance/stiffness matrix N of RUC in this work.

(21) $$N=\sum_{m=1}^{MM}{\langle N\rangle }_{Y}+\sum_{n=1}^{NN}{\langle N\rangle }_{R}+\sum_{l=1}^{LL}{\langle N\rangle }_{Mix}$$

(22) $$\begin{array}{l} {\langle N\rangle }_{I}=\int_{W_{i}}{\left[B\right]}^{T}[{\langle \mathbb{N}\rangle }_{I}][B]dW=\int_{-1}^{1}\int_{-1}^{1}\int_{-1}^{1}{\left[B\right]}^{T}[{\langle \mathbb{N}\rangle }_{I}][B]\det[J]d\xi d\eta d\varsigma \\ \;\;=\sum_{i=1}^{3}\sum_{j=1}^{3}\sum_{k=1}^{3}V_{i}{\bar{V}}_{j}{\tilde{V}}_{k}{\left({\left[B\right]}^{T}[{\langle \mathbb{N}\rangle }_{I}][B]\det[J]\right)}_{\xi =\xi_{i},\eta =\eta_{j},\varsigma =\varsigma_{k}} \end{array}$$

where ${\langle N\rangle }_{I}(I=Y,R,Mix)$ represents the compliance/stiffness related to the corresponding material property matrix $\langle \mathbb{N}\rangle$ of the yarn/resin/mixed element, respectively. MM, NN and LL are the numbers of the yarn/resin/mixed element. [B] is the strain displacement matrix. $\xi_{i}$, $\eta_{j}$ and $\varsigma_{k}$ are the integration points.

Based on the above relationship expression, the relationship between the mean mechanical properties of 3D braided carbon/resin composites and the standard deviation of the corresponding multi-type random characteristics parameters can be obtained through numerical calculation.

## 3. Results and Discussion

### 3.1. Mechanical Properties’ Response of Distortion Yarn

The mechanical properties’ response of the 3D braided carbon/resin composites was analyzed based on the material parameters list in Table 1. The matrix was TDE-86 epoxy resin and the fiber was 12K T700 carbon fiber. In order to further study the influence of yarn squeeze randomness on the mean elastic constants $\langle EC\rangle$ of 3D braided carbon/resin composites, the relationship between yarn mean elastic constants $\langle EC\rangle$ in the material property matrix $\langle \mathbb{N}\rangle$ of Equation (18) and the random parameters mentioned in the second section were analyzed.

**Figure 4:**
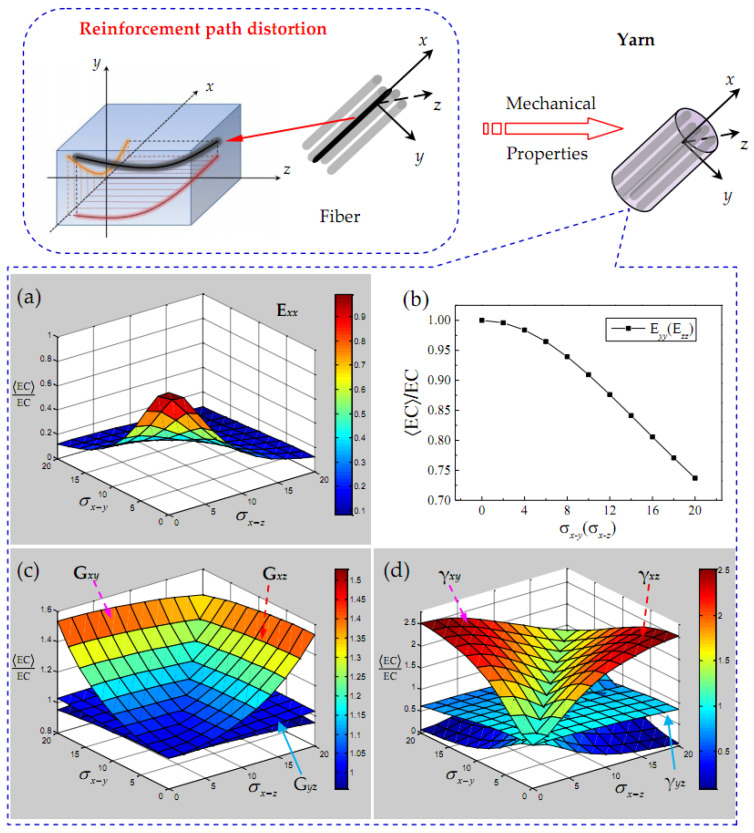
Elastic characteristics of a yarn versus $\sigma_{\alpha_{i}}\left(i=x-y,x-z\right)$ yarn path. (**a**) Elastic modulus E*_xx_*; (**b**) elastic modulus E*_yy_*(E*_zz_*); (**c**) shear modulus G*_xy_*, G*_yz_* and G*_xz_*; and (**d**) poisson’s ratio γ*_xy_*, γ*_yz_* and γ*_xz_*.

Considering the random waviness of the yarn path, the change of the mean elastic constant $\langle EC\rangle$ divided by the ideal elastic constant EC of a yarn is presented in Figure 4. Within the current parameter range, it can be seen that, except for the transverse elastic modulus $E_{yy}$ or $E_{zz}$ of yarn, other elastic constants are seriously affected by $\sigma_{x-y}$ and $\sigma_{x-z}$. The longitudinal elastic modulus $E_{xx}$ and in-plane shear modulus $G_{yz}$ of yarn show the symmetrical characteristics of the changes of $\sigma_{x-y}$ and $\sigma_{x-z}$, and the elastic constants $E_{yy}$, $E_{zz}$, $G_{xy}$, $G_{xz}$, $\gamma_{xy}$ and $\gamma_{xz}$ of the same type also show a certain symmetry, which further reflects the transverse isotropy for yarn. The waviness effect of yarn along each direction will obviously affect the elastic constant in the corresponding direction, but in general, the yarn waviness seems to have a greater impact on Poisson’s ratio $\gamma_{xy}$ and $\gamma_{xz}$. In addition, the out-of-plane shear modulus $G_{xy}$, $G_{xz}$ and Poisson’s ratio $\gamma_{xy}$ and $\gamma_{xz}$ increase with the increase in yarn waviness in the corresponding direction (*x*–*y* or *x*–*z*), but the in-plane shear modulus $G_{yz}$ and Poisson’s ratio $\gamma_{yz}$ are less affected by yarn waviness.

**Figure 5:**
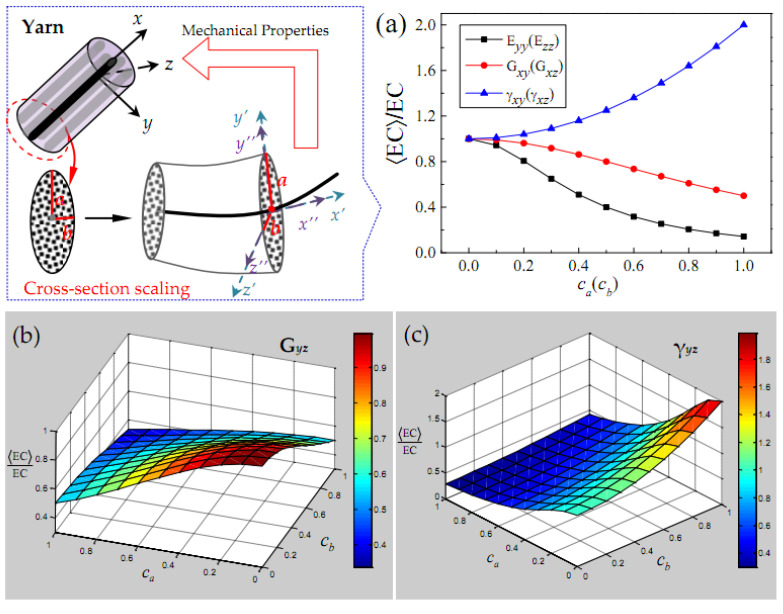
The elastic characteristics of a yarn related to the cross-section shape parameters $c_{i}$$\left(i=a,b\right)$ in the case of only stochastic scaling. (**a**) Elastic constants Eyy (Ezz), Gxy (Gxz) and γxy (γxz); (**b**) shear modulus Gyz; (**c**) Poisson’s ratio γyz.

Considering the random scaling effect of the long and short semi-axis of the elliptical cross-section of yarn, the change in the mean elastic constant $\langle EC\rangle$ divided by the ideal elastic constant EC of yarn is presented in Figure 5. It should be explained that the longitudinal elastic constant $E_{xx}$ of yarn is independent of the shape parameters $c_{a}$ and $c_{b}$, since the random scaling effect of the cross-section only acts on the *y*-axis and *z*-axis. It can be seen from Figure 5a that the transverse elastic modulus $E_{yy}$($E_{zz}$) and the out-of-plane Poisson’s ratio $\gamma_{xy}$($\gamma_{xz}$) are more affected by the yarn cross-section scaling than the out-of-plane shear modulus $G_{xy}$($G_{xz}$). The transverse elastic modulus $E_{yy}$($E_{zz}$), out-of-plane shear modulus $G_{xy}$($G_{xz}$) and out-of-plane Poisson’s ratio $\gamma_{xy}$($\gamma_{xz}$) of yarn are only determined by the change of scaling parameters ($c_{a}$,$c_{b}$) in the corresponding direction. According to Figure 5c,d, the in-plane shear modulus $G_{yz}$ and in-plane Poisson’s ratio $\gamma_{yz}$ are jointly determined by the change of scaling parameters ($c_{a}$, $c_{b}$) in the direction of the long and short semi-axis of the elliptical cross-section.

**Figure 6:**
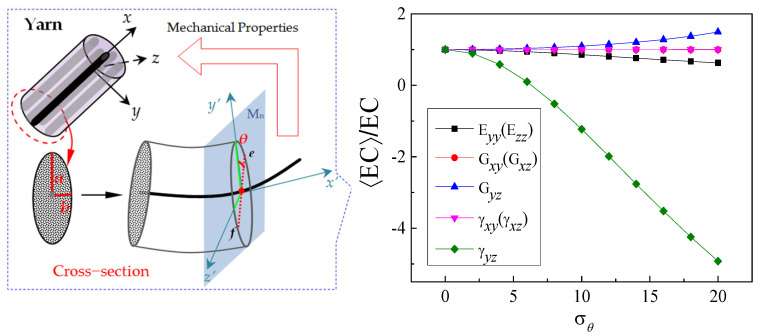
Mechanical properties of a yarn related to the standard deviation $\sigma_{\theta }$ in the case of only stochastic twist.

In addition, the effect of the random torsional effect of a cross-section on the elastic constants of the yarn is also shown in Figure 6.

The out-of-plane shear modulus $G_{xy}$ ($G_{xz}$) and out-of-plane Poisson’s ratio $\gamma_{xy}$($\gamma_{xz}$) of yarn are not sensitive and are negligible to the yarn cross-section twist, and the longitudinal elastic constant $E_{xx}$ of yarn is independent of the standard deviation $\sigma_{\theta }$. With the increase in the cross-section torsion angle, the in-plane shear modulus $G_{yz}$ will gradually increase, while the transverse elastic modulus $E_{yy}$ and $E_{zz}$ show the opposite trend. It can be seen from Figure 6 that the effect of the yarn cross-section torsion on in-plane Poisson’s ratio $\gamma_{yz}$ is more obvious, and it is $\sigma_{\theta }>2.5$.

### 3.2. Mechanical Properties’ Response of 3D Braided Carbon/Resin Composites

#### 3.2.1. Influence of Random Parameters

**Figure 7:**
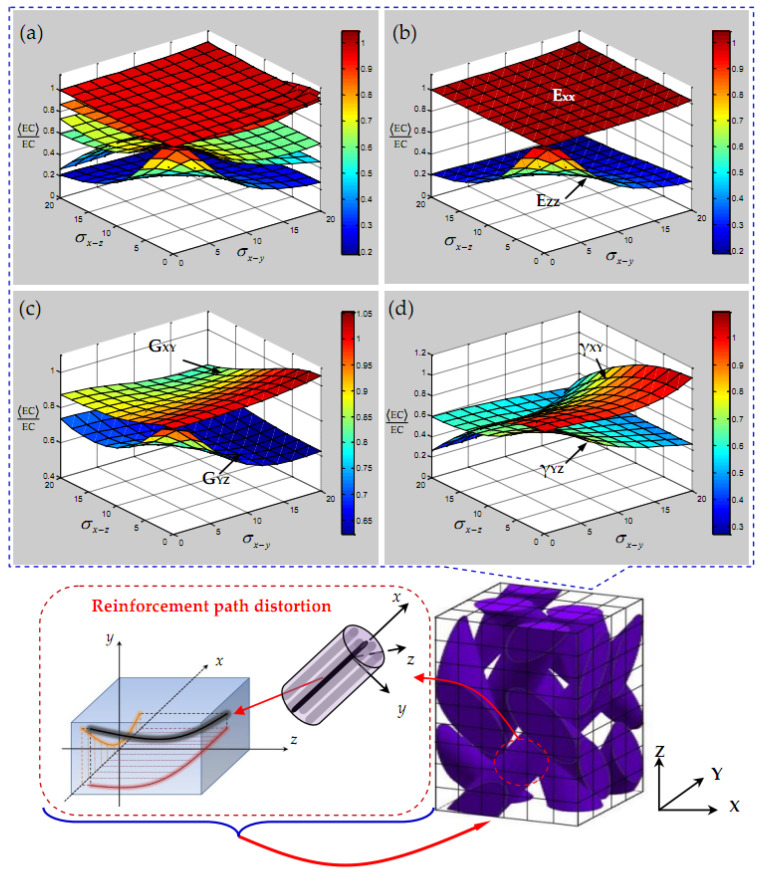
Elastic characteristics of 3D braided carbon/resin composites with a 20° braiding angle versus the standard deviation $\sigma_{x-y}$ and $\sigma_{x-z}$ of a yarn: (**a**) all of the elastic constants, (**b**)elastic modulus, (**c**) shear modulus, (**d**) Poisson’s ratio.

Based on the analysis of yarn elastic constants above, the ratio between the mean elastic constants and the ideal elastic constants of 3D braided carbon/resin composites with a 20° braiding angle is firstly investigated. As shown in Figure 7, the influence of the yarn random waviness effect on the elastic constants of 3D braided carbon/resin composites is presented. It is found from Figure 7a that all of the elastic constants are affected by the random waviness of the yarn path, and the elastic constants for 3D braided carbon/resin composites are affected differently from each other. Among them, it is found from Figure 7b that the transverse elastic modulus E_XX_ increases slightly with the increase in the coupling fluctuation effect in the two planes of the yarn, but the influence of the random fluctuation of the yarn is almost negligible. The longitudinal elastic modulus E_ZZ_ is obviously affected by yarn waviness, but the influence trend of E_ZZ_ gradually slows down with the increase in the standard deviation $\sigma_{x-y}$ and $\sigma_{x-z}$ of a yarn. Specifically, when $\sigma_{x-y}$ or $\sigma_{x-z}>10{}^{\circ}$, as in Figure 7c, it is found that the in-plane shear modulus G_XY_ is less affected by the waviness in the *x*–*y* plane of the yarn, while the out-of-plane shear modulus G_YZ_ is obviously affected by the waviness in both planes of the braided yarn, and the trend tends to be gradual. It is found in Figure 7d that the out-of-plane Poisson’s ratio *γ*_YZ_ shows the same characteristics as E_ZZ_ and G_YZ_, but the in-plane Poisson’s ratio *γ*_XY_ seems to be more obviously affected by the waviness in the *x*–*z* plane for yarn, and shows a sharp downward trend.

**Figure 8:**
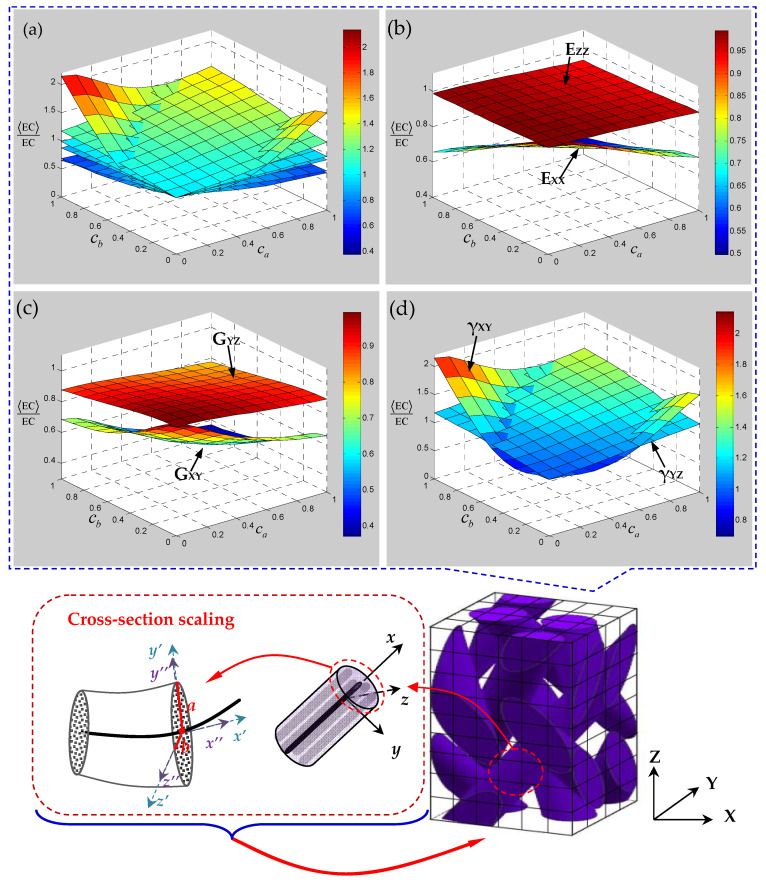
Mechanical properties of 3D braided carbon/resin composites with a 20° braiding angle related to the cross-section shape parameters $c_{a}$ and $c_{b}$ of a yarn: (**a**) all of the elastic constants, (**b**) elastic modulus, (**c**) shear modulus, (**d**) Poisson’s ratio.

On the premise of considering the cross-section random scaling of yarn, the ratio relationship between the mean elastic constants and the ideal equivalent elastic constants of 3D braided carbon/resin composites with 20° braiding angle is given in Figure 8. It can be seen from Figure 8a that, compared with the elastic characteristics of 3D braided carbon/resin composites under the influence of yarn waviness, the random scaling of a yarn cross-section has a much weaker effect on the elastic constants. It is found from Figure 8b–d that the influence of random scaling of a yarn cross-section on the elastic constants E_ZZ_, G_YZ_ and *γ*_YZ_ changes almost linearly, and the change trend is relatively gentle compared with elastic constants E_XX_, G_XY_ and *γ*_XY_. Although the yarn cross-section scaling has little effect on the longitudinal elastic modulus of the yarn, the longitudinal elastic modulus of the 3D braided composite shows a slight decreasing trend under the influences of the transverse elastic modulus of the yarn. In particular, it is found that with the increase in the random scaling degree for the elliptical cross-section, elastic constants E_XX_ and G_XY_ show the characteristics of gradual decline, while Poisson’s ratio *γ*_XY_ shows a trend of overturning.

**Figure 9:**
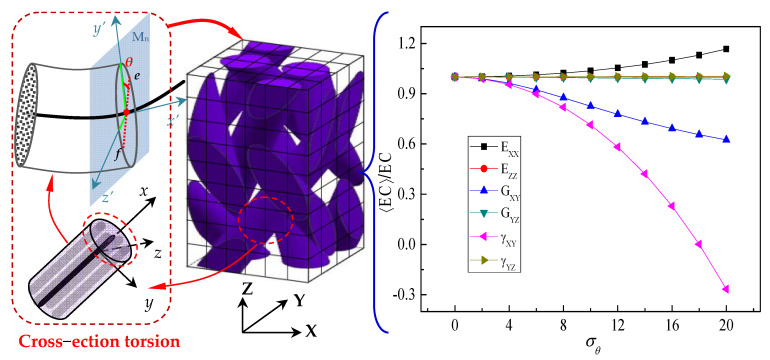
Effect of cross-section distortion characteristic parameters $\sigma_{\theta }$ of a yarn on the mechanical properties of 3D braided carbon/resin composites with a 20° braiding angle.

Moreover, on the premise of considering the random torsion effect of the yarn cross-section, the changed results of the elastic constants of 3D braided carbon/resin composites with a 20° braiding angle are shown in Figure 9. The out-of-plane Poisson’s ratio *γ*_YZ_, out-of-plane shear modulus G_YZ_ and longitudinal elastic modulus E_ZZ_ do not appear to be sensitive to the random section torsion of the yarn. Compared with the elastic constants E_ZZ_, G_YZ_ and *γ*_YZ_, when the standard deviation $\sigma_{\theta }$ is greater than 2.5, the changes of the in-plane Poisson’s ratio *γ*_XY_, in-plane shear modulus G_XY_ and transverse elastic modulus E_XX_ are stronger with the increase in torsion effect for the yarn cross-section. Importantly, the in-plane Poisson’s ratio *γ*_XY_ will fluctuate more violently when the standard deviation $\sigma_{\theta }$ exceeds 10.

#### 3.2.2. Influence of Braiding Angle

Based on the analysis hereinbefore, it can be found that the distortion caused by yarn squeeze will seriously affect the mean elastic constants of 3D braided carbon/resin composites. In order to detect the sensitivity of 3D braided carbon/resin composites with different braiding parameters to the random parameters mentioned above, three braiding angle (20°, 30°, 40°) composites are selected in this section.

**Figure 10:**
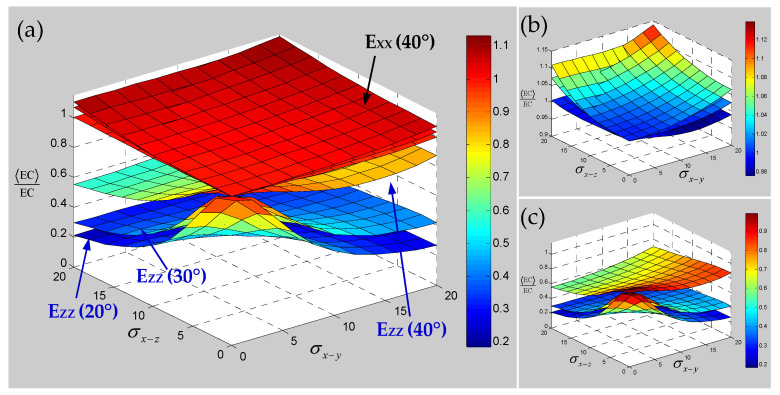
Sensitivity of elastic modulus for 3D braided carbon/resin composites with different braiding angles (20°, 30°, 40°) to the distortion characteristic parameters $\sigma_{x-y}$ and $\sigma_{x-z}$ of a yarn: (**a**) general drawing, (**b**) elastic modulus E_XX_, (**c**) elastic modulus E_ZZ_.

**Figure 11:**
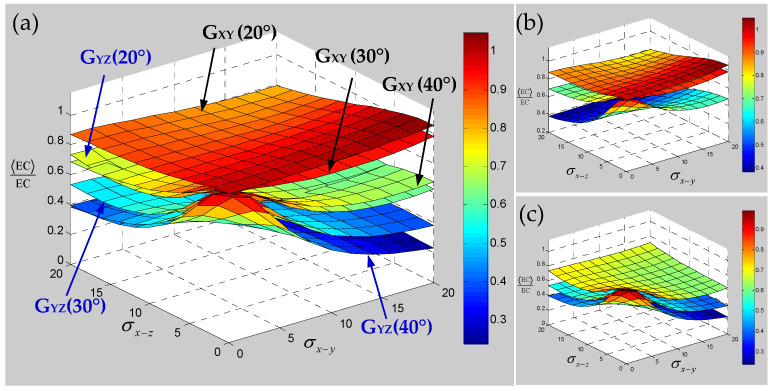
Sensitivity of shear modulus for 3D braided carbon/resin composites with different braiding angles (20°, 30°, 40°) to the distortion characteristic parameters $\sigma_{x-y}$ and $\sigma_{x-z}$ of a yarn: (**a**) general drawing, (**b**) shear modulus G_XY_, (**c**) shear modulus G_YZ_.

**Figure 12:**
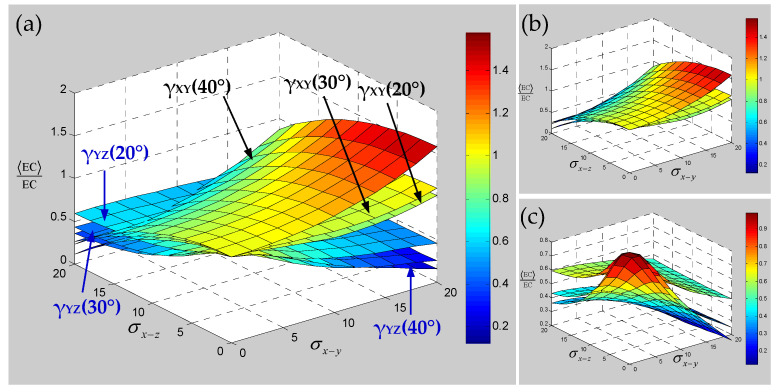
Sensitivity of Poisson’s ratios for 3D braided carbon/resin composites with different braiding angles (20°, 30°, 40°) to the distortion characteristic parameters $\sigma_{x-y}$ and $\sigma_{x-z}$ of a yarn: (**a**) general drawing, (**b**) Poisson’s ratio *γ*_XY_, (**c**) Poisson’s ratio *γ*_YZ_.

It can be seen from Figure 10 that with the increase in the braiding angle, the sensitivity of the transverse elastic modulus E_XX_ to the yarn waviness effect slightly increases, but there is a certain intersection area and the performance is not obvious. The sensitivity of longitudinal elastic modulus E_ZZ_ to the yarn waviness effect is gradually reduced, which indicates that the influence of yarn waviness on the material longitudinal elastic modulus E_ZZ_ will gradually decrease with the increase in braiding angle in the current range. This situation shows that the influence of yarn waviness on the elastic modulus of a 3D braided composite will gradually be offset with the gradual increase in braiding angle. Figure 11 shows the effect of yarn waviness on the shear modulus; it can be seen that the change of in-plane shear modulus G_XY_ on the effect of yarn waviness is more and more obvious with the increase in braiding angle, and the sensitivity to the fiber waviness effect in different planes shows different trends. In contrast to the out-of-plane shear modulus G_YZ_, although the sensitivity also increases gradually with the increase in braiding angle, the sensitivity to the yarn waviness effect in different planes changes more evenly. Compared with the longitudinal or transverse elastic modulus, it can be seen that although the increase in braiding angle can effectively reduce the influence of yarn fluctuation effect on the longitudinal or transverse elastic modulus, it will also increase the sensitivity of the shear modulus. Figure 12 represents the effect of yarn waviness on Poisson’s ratios of 3D braided carbon/resin composites. With the increase in braiding angle, the sensitivity of in-plane or out of plane Poisson’s ratio to yarn waviness effect seems to increase in varying degrees, and the larger the braiding angle is, the stronger the increasing trend is. Based on the above sensitivity analysis of the elastic constants for 3D braided carbon/resin composites with different braiding angles considering the yarn waviness effect, it is found that the influence of the yarn waviness effect on the elastic constants of materials will not be completely eliminated due to the change of braiding angle. At the same time, it also exhibits uncertainty and complexity for the influence of the yarn fluctuation effect on the elastic constants of 3D braided carbon/resin composites.

**Figure 13:**
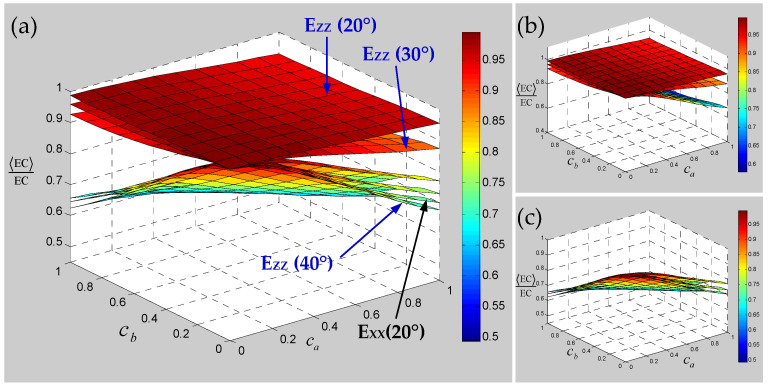
Relevance of elastic modulus of 3D braided carbon/resin composites with different braiding angles (20°, 30°, 40°) to the cross-section shape parameters $c_{a}$ and $c_{b}$ of a yarn. (**a**) general drawing, (**b**) elastic modulus E_ZZ_, (**c**) elastic modulus E_XX_.

**Figure 14:**
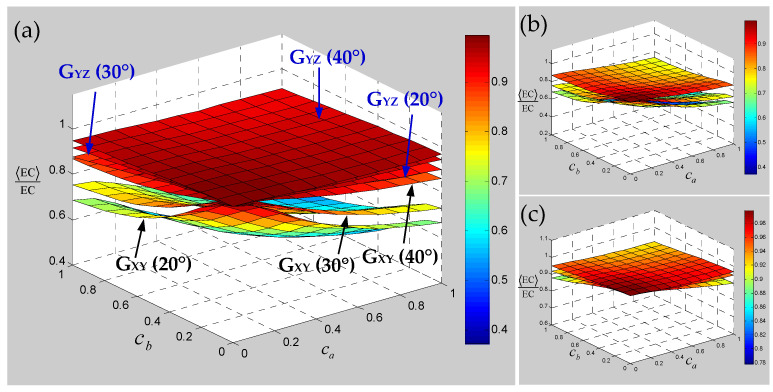
Relevance of shear modulus of 3D braided carbon/resin composites with different braiding angles (20°, 30°, 40°) to the cross-section shape parameters $c_{a}$ and $c_{b}$ of a yarn: (**a**) general drawing, (**b**) shear modulus G_XY_, (**c**) shear modulus G_YZ_.

**Figure 15:**
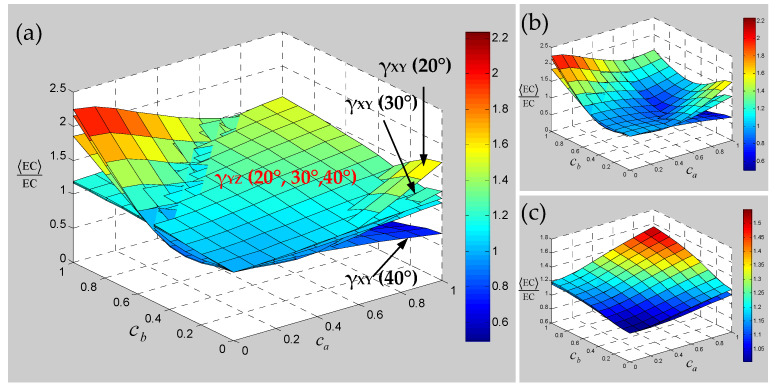
Relevance of Poisson’s ratios of 3D braided carbon/resin composites with different braiding angles (20°, 30°, 40°) to the cross-section shape parameters $c_{a}$ and $c_{b}$ of a yarn: (**a**) general drawing, (**b**) Poisson’s ratio *γ*_XY_, (**c**) Poisson’s ratio *γ*_YZ_.

Numerical results of the sensitivity for the elastic constants of 3D braided carbon/resin composites with different braiding angles to the random scaling effect of a yarn cross-section are presented in Figure 13, Figure 14 and Figure 15. As shown in Figure 13, the change of braiding angle causes the longitudinal elastic modulus E_ZZ_ to be more sensitive to the yarn cross-section scaling than the transverse elastic modulus E_XX_, and the sensitivity of the longitudinal elastic modulus E_ZZ_ to the yarn cross-sectional scaling increases gradually with the increase in braiding angle. However, the sensitivity of the transverse elastic modulus E_XX_ to the scaling effect of the yarn cross-section is not obvious with the increase in braiding angle, and there is a certain intersection area. As shown in Figure 14, the influence of braiding angle change and yarn cross-section scaling on the in-plane and out-of-plane shear modulus of the material is determined. The increase in braiding angle leads to the decrease in the sensitivity of in-plane shear modulus G_XY_ and out-of-plane shear modulus G_XZ_ to the random scaling effect for yarn cross-section. In contrast, the in-plane shear modulus G_XY_ is more sensitive to the random scaling effect of a yarn cross-section under the influence of braiding angle. It also shows that the effect of random scaling for yarn cross-section on the shear modulus will gradually decrease with the increase in braiding angle. As shown in Figure 15, the effect of braiding angle on the elastic constants *γ*_XY_ and *γ*_YZ_ under the scaling effect of yarn cross-section is obviously different. The sensitivity of the out-of-plane Poisson’s ratio *γ*_YZ_ to the scaling effect of the yarn cross-section is very small, and almost negligible due to the change of the braiding angle. The sensitivity of the in-plane Poisson’s ratio *γ*_XY_ to the scaling effect of the yarn cross-section is gradually increased by the change of the braiding angle, and the effect is small when the characteristic parameter ($c_{a}$, $c_{b}$) is less than 0.4. In general, with the increase in braiding angle, the sensitivity of material elastic constants to yarn cross-section scaling effect is slightly weaker than that of yarn path waviness shown in Figure 10, Figure 11 and Figure 12.

**Figure 16:**
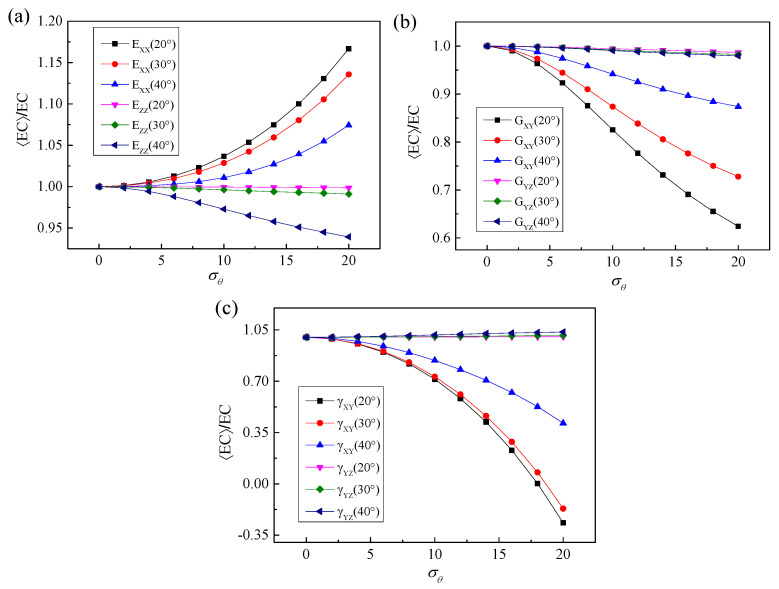
Mechanical properties’ response to 3D braided carbon/resin composites with different braiding angles (20°, 30°, 40°) to the cross-section torsional parameter $\sigma_{\theta }$ of a yarn: (**a**) elastic modulus, (**b**) shear modulus, (**c**) Poisson’s ratios.

Then, the sensitivity of the elastic constants of 3D braided carbon/resin composites with different braiding angles to the torsional effect of yarn cross-section is studied. It can be seen from Figure 16 that the sensitivity for elastic constants with different braiding angles to the random torsion effect of a yarn cross-section has changed significantly and regularly. Among them, the out-of-plane shear modulus G_YZ_ and Poisson’s ratio *γ*_YZ_ of different braiding angles are not sensitive to the torsional effect of the yarn cross-section. With the increase in braiding angle, the sensitivity of transverse elastic modulus E_XX_, in-plane shear modulus G_XY_ and in-plane Poisson’s ratio *γ*_XY_ to the torsional effect of yarn cross-section decreases gradually. By contrast, the longitudinal elastic modulus Ezz shows the opposite sensitive change trend, but compared with E_XX_, G_XY_ and *γ*_XY_, the variation of the braiding angle results in a relatively weak sensitivity for the longitudinal elastic modulus E_ZZ_ to the torsional effect of the yarn cross-section. In general, except for the out-of-plane shear modulus G_YZ_ and Poisson’s ratio *γ*_YZ_, the sensitivity of other elastic constants to braiding angle shows a divergent state after the cross-section torsional standard deviation $\sigma_{\theta }$ of yarn is greater than 2.5.

## 4. Conclusions

The current research reports the effect of multi-type distortion characteristics caused by yarn squeeze on the mechanical properties of 3D braided carbon/resin composites. Firstly, the mechanical properties of yarn are studied by introducing the following three kinds of random parameters, including yarn path, yarn cross-section size and yarn cross-section torsion. It is found that the numerical results can show the transverse isotropic characteristics of yarn, and the influence of multi-type microscopic distortion on the mechanical properties of yarn is obvious. The influence of path waviness on the transverse mechanical properties $E_{yy}$, $E_{zz}$, $G_{yz}$ and $\gamma_{yz}$ of yarn is much less than that on the longitudinal properties $E_{xx}$, $G_{xy}$, $G_{xz}$, $\gamma_{xy}$ and $\gamma_{xz}$. In addition to Poisson’s ratio $\gamma_{xy}$, the effect of path waviness on other elastic moduli of yarns tends to be stable with the increase in yarn waviness standard deviation $\sigma_{x-y}$ and $\sigma_{x-z}$, and the effect of yarn waviness on the elastic constants of 3D braided composites presents the basic characteristics consistent with that of yarns. Yarn cross-section scaling has a great influence on other elastic constants of yarn, except the longitudinal elastic modulus $E_{xx}$, especially Poisson’s ratio. At the same time, for 3D braided composites, except for in-plane Poisson’s ratio $\gamma_{XY}$, the elastic constants of other materials are similar to the characteristics of yarn mechanical properties under the influence of cross-section scaling. In addition to the in-plane shear modulus and transverse elastic modulus, the basic characteristics of yarn mechanical properties under the influence of a section torsion effect are consistent with those of braided composites. In general, cross-section scaling and torsion have great influence on the transverse mechanical properties of yarn, especially the in-plane Poisson’s ratio $\gamma_{yz}$. The dispersion of the mechanical properties of 3D braided carbon/resin composites caused by yarn random waviness is greater than that of cross-section scaling and torsion. The variation of yarn cross-section size and torsion will lead to more serious fluctuations of transverse properties than longitudinal properties of 3D braided carbon/resin composites. In addition, it is also found that 3D braided carbon/resin composites with different braiding angles have different sensitivity to the yarn distortion characteristics parameter.

## Figures and Tables

**Table 1:** Mechanical property parameters of fiber and matrix.

Component	Modulus (GPa)				*γ*_12_
	*E*_11_	*E*_22_	*E*_12_	*E*_23_	
TDE-86 resin	3.45	3.45	—	—	0.35
T700-12K fiber	215.6	17.21	12.92	9.3	0.3

## Data Availability

Not applicable.
